# Capture-recapture methodology to study rare conditions using surveillance data for fragile X syndrome and muscular dystrophy

**DOI:** 10.1186/s13023-017-0628-y

**Published:** 2017-04-21

**Authors:** Michael G. Smith, Julie Royer, Joshua Mann, Suzanne McDermott, Rodolfo Valdez

**Affiliations:** 1 Department of Health Services Management and Policy, East Tennessee State University, Johnson City, TN USA; 2 Revenue and Fiscal Affairs Office, Health and Demographics Section, Columbia, SC USA; 3 Department of Preventive Medicine, University of Mississippi Medical Center, Jackson, MS USA; 4 Department of Epidemiology and Biostatistics, University of South Carolina, Columbia, SC USA; 5 Centers for Disease Control and Prevention, National Center for Birth Defects and Developmental Disabilities, Atlanta, GA USA

**Keywords:** Capture-recapture, Muscular dystrophy, Fragile X syndrome, Passive surveillance

## Abstract

**Background:**

Rare conditions can be catastrophic for families and the implications for public health can be substantial. Our study compared basic surveillance through active medical record review with a linked administrative data file to assess the number of cases of two rare conditions, fragile X syndrome (FXS) and muscular dystrophy (MD) in a population.

**Methods:**

Two methods of data collection were used to collect information from five counties comprising two standard metropolitan statistical areas of South Carolina. The passive system relied mostly on health claims data using ICD-9 CM diagnostic codes. The active system relied on a nurse abstracting records from a list of all licensed physicians with specialties in neurology, orthopedics, and genetics.

**Results:**

There were 141 FXS cases and 348 MD cases that met the case definitions using active surveillance. Additional cases were found for both conditions but they were determined to not be true cases. After linking the actively collected MD and FXS cases to passive datasets, we found that the estimated total numbers of cases were similar to using capture-recapture analysis; the positive predictive values for cases identified in the passive system were 56.6% for MD and 75.7% for FXS.

**Conclusions:**

Applying capture-recapture methods to passively collected surveillance data for rare health conditions produced an estimate of the number of true cases that was similar to that obtained through active data collection.

## Background

There are many rare conditions that first manifest symptoms in childhood and persist into adulthood. It is often difficult to effectively and efficiently estimate the number of cases of these rare conditions in a specific area. Some methods, such as reportable condition registries, may require legislation or other state-level policy, while methods such as medical record abstraction are costly and time-intensive. It is our intention to demonstrate a methodology that uses administrative data to document rare conditions, when resources are not available to conduct active case finding. This paper uses two rare conditions that primarily manifest in males, muscular dystrophy (MD) and Fragile X syndrome (FXS), to test an algorithm that applies capture-recapture methods to linked administrative datasets in order to estimate the number of cases of each condition receiving care in a five county area of South Carolina. The reported prevalence in the literature is approximately 1/4,000 males for FXS to 1/5,000 males for MD [1–3].

The expectation that linked administrative records, or passive surveillance systems, are reliable sources for estimating the true prevalence for health conditions, has been questioned by many public health professionals and policy makers [4]. Clearly the low cost of passive surveillance is attractive, if the data are reasonably accurate. Concerns that have been raised include anticipated inaccuracy associated with imprecision of codes used in both clinical billing and public agency data. Thus, active surveillance, which includes professional review of records to validate case status, is preferred when sufficient funding is available. However, despite reservations about the accuracy of passive surveillance, when resources are limited, the use of administrative and claims data has been a longstanding practice [5–7]. Passive surveillance may be particularly useful for rare conditions, where the resource demands for establishing effective active surveillance systems may be very large in relation to the number of cases identified.

There is limited research on the validity of passive surveillance compared to active surveillance (professional record review). One study compared active chart review with a hospital database found that inter-database agreement rates varied from relatively high agreement for common conditions such as diabetes (k = 0.83), good agreement for myocardial infarction and chronic renal failure (k = 0.52-0.62), and low agreement for symptoms such as hyperlipidemia [8] The validity of passive surveillance approaches for rare conditions has not yet been established.

An important issue related to passive surveillance is deciding on a case definition. When conditions are rare and estimates of prevalence are wanted some researchers have accepted one code in the primary or secondary diagnosis field among people who were enrolled in the insurance plan for a minimum of two years [9]. One of the strategies used to improve the accuracy of use of medical insurance claims is to use all the fields for diagnoses and the coding from multiple visits [10–12]. Another strategy is to require at least two records from each source to define a case [13, 14]. Other algorithms for case identification require coding for diagnostic testing or treatment, or at least one hospitalization with the code [15]. Others have required a linkage with a second administrative data source to verify a disability [6].

In our study of two rare conditions we used a linked administrative data file that included billing data from a number of insurers as well as data from agencies that provided services for people with disability. All cases in the population may not be covered by one of these insurance providers or receive services from the agencies included in the passive dataset, therefore a capture-recapture algorithm was used to estimate the total number of cases. The primary objective of this study was to determine whether data from multiple passive sources could be used to accurately estimate the number of individuals with rare, lifelong conditions that frequently onset in childhood using capture-recapture methods. Therefore, an estimated number of people with FXS and MD was calculated from passive data sources and compared to the number of cases obtained through active data collection. Additionally, the active and passive datasets were linked to examine how well these autonomous systems identified the same individuals as cases. It is not expected that capture-recapture methods will aid in identifying the correct individual cases, but, instead accurately estimate the total number of cases.

## Methods

### Data collection

Two methods of data collection were used for this project: a passive data system whereby data about cases of FXS and MD statewide were ascertained through the linkage of multiple administrative data systems and an active data collection system whereby data about cases of FXS and MD were abstracted directly from medical records from medical practices that serve individuals with the two conditions. The passive system was statewide and the active surveillance was conducted in five target counties. The data processes were conducted independently without records identified in the passive system being referred to the active system or vice versa. Both approaches were conducted by state agencies; the passive data system was completed by the Health and Demographics Section of the South Carolina Office of Revenue and Fiscal Affairs (RFA) and the active data collection was completed by Maternal and Child Health Bureau at the South Carolina Department of Health and Environmental Control (DHEC). DHEC is the state health department and RFA serves as a central repository for health and human service data in South Carolina.

The passive data system linked data from the following sources to identify potential cases of FXS and MD statewide: uniform billing hospital discharge including inpatient hospitalizations and emergency department visits, a private insurer, Medicaid data and a disability service agency. Data from inpatient admissions and emergency department visits for all hospitals operating in South Carolina were included in the uniform billing hospital discharge data. Every diagnosis code given at each hospital visit was included in the data analyzed. In some instances there were over 12 diagnosis codes given for a single hospital admission, all of which were used in this study if they indicated MD or FXS. The private insurer data utilized in this study covered state government employees (including teachers) statewide. The data from the disability service agency included voluntary registry data for individuals with FXS or MD who registered for services. All data with service dates from 1996-2012 were included in this linked passive surveillance dataset. This 17 year surveillance period increases the likelihood that an individual with FXS or MD will receive services through one or more of the passive surveillance data sources, as utilization of these services depends greatly on the severity and progression of the condition.

We used the International Classification of Diseases, 9^th^ revision, Clinical Modification (ICD-9-CM) code 759.83 to identify potential FXS and codes 359.0 (congenital hereditary muscular dystrophy), 359.1 (hereditary progressive muscular dystrophy), and 359.21 (myotonic muscular dystrophy) to identify potential MD cases from health claims and an indicator variable from non-health claims. This passive data linkage process likely resulted in an overestimate of true number of FXS and MD cases statewide, since it is possible that suspected cases for whom confirmatory tests for FXS or MD were ordered by the physician had negative results. When an individual had only one code for FXS or MD this was most likely the case.

For the active data collection system, each neurology, orthopedics, or genetics physician practice located within the five target counties was sent a letter explaining the surveillance goal of the project, DHEC’s public health authority to access the medical records, and the liability protection afforded the practice in the release of the information by South Carolina state law. Each letter was followed-up with a call to the practice to determine whether the practice had current or past patients with FXS or MD, and, if so, an appointment was made for a DHEC nurse abstractor to visit the practice to collect the relevant data on each case. Patients were considered to be cases in the active data collection system if there was a positive genetic test or a clear diagnosis as a case from a physician in one of the included specialties. The five target counties were in two standard metropolitan statistical areas (SMSAs).

The actively- and passively-collected data were combined to estimate the number of true cases of FXS and MD statewide through capture-recapture methodology. Data usage approvals were obtained from participating organizations from which the data originated. Data collection was conducted in accordance to prevailing ethical principles and approved by the DHEC Institutional Review Board. All data linkages and analyses were performed at RFA and aggregate results were provided to investigators. The number of cases presented in this paper represent unique individuals and not the number of times a diagnosis code is used. A proprietary unique identifier generation system is utilized by RFA to identify individuals in each passive data source to allow for appropriate individual-level linkage across data sources. This same unique identifier system was applied to the active surveillance data to allow for the individual-level linkage of the active and passively collected data for this analysis.

### Population

The population under study is described with respect to age, race, and whether care was received in one of the active surveillance counties in Table 1. Demographic variables available to describe the population are limited to those common to all passive surveillance sources. For MD and FXS all cases in the active surveillance system received care in the active surveillance counties by definition. Among individuals with an MD ICD code statewide, 43.2% received care in an active surveillance county. Among individuals with an FXS ICD code statewide, 29.1% received care in an active surveillance county. For both MD and FXS the age distribution between the actively collected cases and the passively collected cases were similar. However, a greater percentage of MD and FXS cases identified in the active data system had a race other than white or black or did not have a race indicated in the medical record.

**Table 1:** Demographic characteristics of muscular dystrophy and fragile X syndrome cases reviewed from active and passive surveillance data sources

	Muscular Dystrophy				Fragile X Syndrome			
	Active *n* = 384		Passive *n* = 3305		Active *n* = 141		Passive *n* = 795	
Demographic Characteristic								
Age Group (in years)	n	%	n	%	n	%	n	%
0 to 9	26	6.8%	286	8.7%	12	8.5%	80	10.1%
10 to 19	57	14.8%	477	14.4%	35	24.8%	169	21.3%
20 to 29	62	16.1%	389	11.8%	31	22.0%	202	25.4%
30 to 39	59	15.4%	338	10.2%	23	16.3%	114	14.3%
40 to 49	37	9.6%	405	12.3%	12	8.5%	94	11.8%
50 to 59	53	13.8%	483	14.6%	14	9.9%	57	7.2%
60 or older	90	23.4%	927	28.0%	14	9.9%	79	9.9%
Race								
White	262	68.2%	1998	60.5%	66	46.8%	433	54.5%
Black (African American)	75	19.5%	805	24.4%	57	40.4%	286	36.0%
Other/Unknown	47	12.2%	97	2.9%	18	12.8%	76	9.6%
Receiving Care in Active Counties	384	100.0%	1428	43.2%	141	100.0%	231	29.1%

### Capture-recapture analysis

Capture-recapture methods were applied to passive data sources to develop an estimate of the number of cases for FXS and MD for comparison to numeric estimates from the active data collection system. The log-linear estimation method of capture-recapture analysis was employed to estimate the number of cases of FXS and MD in South Carolina [16, 17]. Capture-recapture counts the number of cases that appear in more than one of the data sources and using this information to estimate the number of cases that do not appear in any of the data sources.

Capture-recapture analyses apply probabilistic estimation techniques to multiple incomplete lists of cases to estimate the number of cases in the underlying population. These methods rely on assessing the number of individual cases that are identified on more than one list and assessing the independence of the lists. The technique employed in this analysis (log-linear estimation) uses log-linear Poisson regression models to account for dependence by adjusting for the frequency with which individuals appear on more than one list. These models are then used to predict the number of unobserved cases.

If three incomplete lists of cases are used, the number of cases identified only on the first list can be denoted as *Z*_*100*_. Similarly, the number of cases identified only on the second list can be denoted as *Z*_*010*_ and the number of cases identified on both the second and third lists can be denoted as *Z*_*011*_ and so on. Then, *Z*_*000*_ represents the number of cases in the population unobserved on any of the incomplete lists. Each of these observed numbers of cases appearing on each combination of lists, *Z*_*ijk*_, can be predicted with a log-linear model using information from the number of cases observed on the other combination of lists using Eq. (1). This results in predicted values (*Ẑ*_001_, *Ẑ*_010_, *Ẑ*_100_, …) for each observed number cases. Then, the number of unobserved cases can be estimated based on the estimates from the observed cases using Eq. (2). This analysis was conducted using SAS 9.4 (SAS Institute, Cary, NC) under the capture-recapture assumption that each data source was indpendent. Therefore, interaction among the data sources was not assessed. Additional details about this capture-recapture methodology can be found elsewhere [16, 17].

1 $$ \begin{array}{l} logE\left({Z}_{ijk}\right)= u+{u}_1 I\left( i=1\right)+{u}_2 I\left( j=1\right)+{u}_3 I\left( k=1\right)+{u}_{12} I\left( i= j=1\right)+{u}_{13} I\left( i= k=1\right)\\ {}\kern4em +{u}_{23} I\left( j= k=1\right)+{u}_{123} I\left( i= k= k=1\right)\end{array} $$

2 $$ \raisebox{1ex}{${\widehat{Z}}_{000}={\widehat{Z}}_{001}{\widehat{Z}}_{010}{\widehat{Z}}_{100}{\widehat{Z}}_{11}$}\!\left/ \!\raisebox{-1ex}{${\widehat{Z}}_{110}{\widehat{Z}}_{101}{\widehat{Z}}_{011}$}\right. $$

One important assumption of capture-recapture methods is that each data source includes an underestimate of the total population of cases. Since codes are used for tests performed to both confirm and to rule out a diagnosis, our sample violates the assumption that the codes represent an underestimate of the true number of cases. Therefore, in this study we sought to pare down the potential FXS or MD cases identified through the passive system so that they represent a subset of true cases. We did this using a step-wise approach with the following algorithm:

1. Start with the total number of cases identified by at least one ICD-9-CM code in the passive dataset,
2. Restrict to cases with at least one ICD-9-CM code from a facility located in the five target counties,
3. Restrict to cases with at least one in-patient ICD-9-CM code or at least two outpatient ICD-9-CM codes from a facility located in the five target counties,
4. Restrict to cases with at least one in-patient ICD-9-CM code or at least two outpatient ICD-9-CM codes in a facility located in the five target counties where at least one of the diagnoses was made by a neurologist, geneticist, or developmental pediatrician.

This algorithm is applied until the analyst is confident that the number of cases included represents an underestimate of the number of true cases in the given population. In the present analysis we compare the number of passively identified cases present at each step of the algorithm with the number of cases identified using active surveillance, terminating the algorithm when the number of passively identified cases is less than the number of actively identified cases. In other situations a complete list of actively identified cases would likely not be available. In these instances we recommend using an estimated number of cases based applying a published prevalence estimate to the population under study. After this restriction was made and the total number of cases in the passive system was an underestimate of the total number of cases in the active system, log-linear estimation capture-recapture methods were applied to estimate the number of cases [18, 19].

The number of cases estimated through the passive analysis was compared to the number of cases abstracted through the active data collection system, considering the active data collection to be the ‘gold-standard’.

## Results

There were 384 MD cases confirmed using active surveillance among practices located in the five target counties. Additionally, there were 1,683 records identified as potential MD cases by the practices that were abstracted, but determined to not be true cases upon clinical review. There were 141 FXS cases confirmed using active surveillance among practices located in the five target counties and 72 additional records were abstracted but determined to not be cases.

From the passive system, the number of sources on which each MD and FXS case is found is displayed in Table 2. We are unable to publicly identify the number of cases identified by each combination of sources due to the restrictions outlined in the data use agreement with RFA that provides access to the passive data. The number of potential cases identified at each step of the passive data restriction algorithm is presented in Table 3.

**Table 2:** Number of data sources on which each passively identified muscular dystrophy and fragile X syndrome case is found

	# of Sources	n	%
Muscular Dystrophy	1	2275	68.8%
	2	872	26.4%
	3 or more	158	4.8%
Fragile X Syndrome	1	586	73.7%
	2	147	18.5%
	3 or more	62	7.8%

**Table 3:** Number of cases identified in active and passive surveillance systems at each step of the passive data restriction algorithm

Passive Data Restriction Algorithm Step	Muscular Dystrophy		Fragile X Syndrome	
	Cases Identified - Active	Cases Identified - Passive	Cases Identified - Active	Cases Identified - Passive
1. Cases with at least one ICD-9-CM code	384	3,305	141	795
2. Cases with at least one ICD-9-CM code given in a facility in target counties	384	1,428	141	231
3. Cases with at least one in-patient ICD-9-CM code or at least two outpatient ICD-9-CM codes given in a facility in target counties	384	990	141	134^a^
4. Cases with at least one in-patient ICD-9-CM code or at least two outpatient ICD-9-CM codes given in a facility in target counties where at least one of the diagnoses was made by a neurologist, geneticist, or developmental pediatrician	384	375^a^	141	50

^a^Indicates the step at which the number of cases identified through the passive system is an underestimate of the cases identified through the active system

For MD, the passive dataset does not produce a lower number of cases than the number identified in the active dataset until step 4 in the data restriction algorithm (passive *n* = 375, active *n* = 384). For FXS, the passive dataset does not produce a lower number of cases than the number identified in the active dataset until step 3 (passive *n* = 134, active *n* = 141).

When capture-recapture analysis is applied to the passively collected estimate for the number of MD cases achieved at step 4 of the data restriction algorithm, an additional group of 40 cases is estimated. This results in a total of 415 MD cases estimated through applying a capture-recapture process to the passively collected data, compared to 384 cases that were collected through active surveillance in the same geographic area (Table 4).

**Table 4:** Results of capture-recapture analysis to estimate the total number of true cases using only passive data

Muscular Dystrophy				
Population	Cases in Passive Dataset	Additional Cases Estimated by Capture-Recapture	Total Estimated Cases from Passive Data	Total Cases Observed in Active Data
Cases with at least one in-patient ICD-9-CM code or at least two outpatient ICD-9-CM codes given in a facility in target counties where at least one of the diagnoses was made by a neurologist, geneticist, or developmental pediatrician	375	40	415	384
Fragile X Syndrome				
Population	Cases in Passive Dataset	Additional Cases Estimated 2by Capture-Recapture	Total Estimated Cases from Passive Data	Total Cases Observed in Active Data
Cases with at least one in-patient ICD-9-CM code or at least two outpatient ICD-9-CM codes given in a facility in target counties	134	6	140	141

Similarly for FXS, Table 4 shows that an additional six cases were estimated through capture-recapture analysis of the passively collected estimated number of cases after applying the data restriction algorithm. This results in a total of 140 estimated FXS cases through passive data collection supplemented by capture-recapture analysis, compared to 141 cases collected through active surveillance.

After linking the 384 actively collected MD cases and 141 actively collected FXS cases to their respective passive datasets, we found that, despite the fact that the estimated total numbers of cases were reasonably similar to the numbers estimated using capture-recapture analysis, the cases used to develop the estimates did not correspond for the most part to the cases identified through active surveillance. That is, most of the actively identified cases were not identified by the passive system, producing a sensitivity of 20.1% and 39.7% for MD and FXS, respectively (Table 5).

**Table 5:** Sensitivity, Specificity, and Positive Predictive Value of passive dataset after linking to active dataset

Population	Sensitivity	Specificity	Positive Predictive Value
Muscular Dystrophy			
Cases with at least one in-patient ICD-9-CM code or at least two outpatient ICD-9-CM codes given in a facility in target counties where at least one of the diagnoses was made by a neurologist, geneticist, or developmental pediatrician	20.1%	96.5%	56.6%
Fragile X Syndrome			
Cases with at least one in-patient ICD-9-CM code or at least two outpatient ICD-9-CM codes given in a facility in target counties	39.7%	75.0%	75.7%

As might be expected, the passive system performed better when identifying non-cases in the active system, with specificities of 96.5% for MD and 75.7% for FXS. The positive predictive values for cases identified in the passive system were 56.6% for MD and 75.7% for FXS.

## Discussion

Active surveillance through medical record abstraction is time consuming and costly. Passive surveillance for rare conditions that are identified simply searching for diagnosis codes can lead to over-documentation in claims databases. This over-documentation may lead to an overestimation of cases for rare conditions and troubling implications for health services research. The objective of this project was to refine the numeric estimates of two rare conditions by applying capture-recapture methodology to passively collected data.

A primary obstacle for applying capture-recapture methods to passively collected data is that these methods assume that multiple sources each have incomplete data on the number of true cases. In reality, with most claims data sources the number of cases may be overestimated because diagnostic coding is applied to both confirm or to rule out a diagnosis, through misdiagnosis, and when a condition is suspected but unconfirmed. In this study, the cases identified through active medical record abstraction were used as the true number of cases. To get a better estimate from the passive system we proposed first restricting to cases with at least one in-patient diagnostic code or two outpatient diagnostic codes and then, if necessary, restricting to diagnoses made by specialists, who are most likely to diagnose true cases.

It should be noted that this study focused on individuals receiving care for FXS or MD in the five county geographic area covered by active data collection. It is probably equally important to estimate the true cases by county of residence, using active and passive datasets, however, doing so would require using a subset of the cases collected by the active surveillance system (restricted to individuals residing in the five county area).

Applying this data restriction algorithm successfully led to underestimates for both MD and FXS. After this restriction was made, applying capture-recapture analysis resulted in a slight overestimate of the number of MD cases and a very close estimate to the number of FXS cases collected through active surveillance. These estimated numbers of cases seem to be reasonable and indicate that restricting passively collected data and then applying a capture-recapture approach would be much more efficient than conducting active data collection to arrive at these figures.

Of course, in practice an actively collected dataset would likely not be available to apply the data restriction algorithm against, as having an actively collected surveillance data would eliminate the need for linking passive datasets for obtaining an estimated number of cases. In this case, we recommend using prevalence estimates from relevant literature to provide a reasonable number of cases to use in applying the data restriction algorithm. This will allow for the use of the best available prevalence estimates from the literature, with local data adding additional context and information to provide an improved case volume estimate. In the absence of actively collected data, which again would likely be the case in application, combining the capture-recapture estimate and the most appropriate prevalence estimate from relevant literature may provide a suitable range for true cases in an area.

Upon linking the actively collected data to the passively collected data we found that there was little concordance between the actively identified cases and the passively identified cases. This lack of concordance between the active data system and the cases used in the passive capture-recapture estimate is not unexpected. The purpose of applying a capture-recapture algorithm is to estimate the correct number of cases and not to identify “true” cases. The lack of concordance between the actively identified cases and the passively identified cases serves to illustrate that assuming that cases identified through passively collected administrative data using the methods presented in this paper should not be assumed to be “true” cases. A different methodology for identifying a subset of cases from passively collected administrative data that are likely to be “true” cases has been developed and presented elsewhere [20]. Furthermore, a majority of the cases identified in the active surveillance system were also identified in the passive surveillance system (68.2% of actively identified MD cases were found in the passive data system and 69.5% of actively identified FXS cases were found in the passive system), but many of these cases were among those that were dropped when the data restriction algorithm was applied. This was considered to be an acceptable trade-off because the intent of this analysis was to estimate the number of cases in the five-county area and not to identify a subset of passively identified cases that are “true” cases.

It is reasonable to expect that a number of cases could be identified in the active data surveillance system but not be present in the passive data system. For example, there could be a substantial number of MD and FXS cases covered by private insurance through a non-state government employer that have manifested in childhood and received a diagnosis in a neurology office but do not have symptoms severe enough to result in hospitalization or utilization of disability agency services. Similarly, it may not be appropriate to assume that “cases” meeting the strict definition used for passive surveillance in our study but not found by active surveillance are in fact “false positives.” Some of these cases may in fact be true cases that were not treated in the practices reviewed; for example, individuals may travel outside the target counties to receive specialty care related to their condition, or they may lack adequate health insurance coverage and therefore receive their health care in hospital emergency departments or other settings not included in our active surveillance approach [21].

Muscular dystrophy may be an especially difficult set of conditions to correctly identify through passive data collection. As described in Table 3, there were 3,305 potential cases of MD identified through the ICD-9 CM codes specific to MD, while only 384 cases were identified through active data collection. The ICD-9 CM used for MD diagnoses also capture other, related neuromuscular conditions. Therefore, for rare conditions like MD with a clinically complex phenotype and non-specific billing codes, true case identification through passively collected administrative data sources may be less feasible than for other conditions with more specific clinical markers and well-defined billing codes.

Finally, it should be stressed that our choice of criteria for identifying cases in the passive surveillance was not based solely on the desire to maximize sensitivity, specificity and positive predictive value but stipulated that the resulting cases would underestimate the true prevalence. It may be that other case definitions would be preferable to optimize the accuracy of passive data for identifying cases. Identifying these case definitions should be considered in future research.

## Conclusions

In conclusion, the findings of this study suggest that capture-recapture methods may be useful for the purposes of estimating the number of cases of rare conditions in a defined population where administrative data are available, but active data collection is unfeasible. This approach would require beginning with a reasonable estimate of the number of cases for the target population, perhaps based on prevalence estimates from the literature adjusted for the demographic characteristics of the target population. The capture-recapture method provides a framework that will help researchers using big data learn how to improve their estimates.

## Abbreviations

DHEC: Department of Health and Environmental Control
FXS: Fragile X syndrome
ICD-9-CM: International classification of diseases, 9^th^ revision, clinical modification
MD: Muscular dystrophy
RFA: Office of Revenue and Fiscal Affairs
SMSAs: Standard metropolitan statistical areas
